# Experiences of Game-Based Learning and Reviewing History of the Experience Using Player's Emotions

**DOI:** 10.3389/frai.2022.874106

**Published:** 2022-07-15

**Authors:** Kaoru Sumi, Shusuke Sato

**Affiliations:** Future University Hakodate, Hakodate, Japan

**Keywords:** game-based learning, emotions, facial expression, operational history, affective computing, story generation

## Abstract

In this paper, we discuss whether the history of a learning experience, containing action and emotion information, is useful for review of the experience in game-based learning using virtual space. We developed a game-based story generation system that automatically generates scripts in real time by using a player's emotions and actions. The system has two functions: a game-based experiential learning environment and automatic story generation. The system provides the player with a virtual world and a virtual tool operated by using a hand controller and a display. The system recognizes the player's real-time emotions through facial expressions, and it outputs reactions based on these emotions and actions *via* a knowledge-based system when the player operates the tool. Then, it outputs scripts based on the emotions and the history of actions. We evaluated the system by conducting experiments with university students as subjects. As a result, subjects found the stories generated by this system interesting because they were based on the player's experience in the game and used the player's behavioral history and emotions. If we consider this as a record of the learning experience, the learning history is an impressive record accompanied by emotions. Thus, historical information that records the actions and emotions of the learner in real time is considered effective because it allows the learner to recall his or her own experiences after the game experience. The results suggest that the historical information, including the learner's real-time actions and emotions, is helpful for review in learning. There is a possibility that experiential learning through games using virtual spaces, such as the one used in this study, will become widespread in the future. In such cases, it will be necessary to examine the learning effects of using historical information with emotions. Therefore, we believe that the results and discussions in this study will be useful for experiential learning using virtual spaces.

## Introduction

This paper presents a system that combines automatic story generation with game-based experiential learning. The system automatically generates a story based on the user's game-based learning experience and its history.

Story generation has typically been performed using text until now (Dehn, 1981; Sousa, 2000; León and Gervás, 2014; Eger et al., 2015; Kaptein and Broekens, 2015; Pérez y Pérez, 2015). For example, Dramatica (Anne and Huntley, 2004), a script creation tool, supports creation of stories by inputting character dynamics, plot dynamics, and static plot points in a text format. Similarly, Dramatis (Neill and Riedl, 2016) infers a reader's emotions by analyzing the behavior of the characters in a story. Facade is an interactive drama in virtual space (Mateas and Stern, 2003), which can express a player's attitudes and emotions by text during a story through interaction between the player and the system.

We believe that a livelier story can be created using emotions. Recent years have seen the development of story generation systems that use emotions. For example, one system generates a story by changing the emotions preset in non-player characters (NPCs) according to their interactions (Chang and Soo, 2009). It seems, however, that previous studies (Hernandez et al., 2014) could not effectively capture real-time emotions, including subconscious emotions.

One previous consumer video game (Booth, 2009) involved the player using a biosensor to obtain vital data on the degree of tension, from which the enemy's appearance pattern was adjusted accordingly. In addition, other studies (Cavazza et al., 2009; Yannakakis and Togelius, 2011; Gilroy et al., 2012; Lopes et al., 2015) have captured emotions by using biosensors and user reactions, enabling object placement, mapping, and game design in stage creation. There is almost no previous research, however, on emotion-based automatic story generation designed to capture real-time player emotions during gameplay, with adaptation of the script accordingly.

Because many traditional narrative generation systems focus on content from a third-person viewpoint and do not consider emotion-based stories from a first-person viewpoint, we instead propose a different approach. Specifically, we propose a system that can develop content and generate more personal stories by using the user's own real-time emotions and actions from a first-person viewpoint. The system is novel in that it can capture and use real-time emotions.

The system has two functions: a game-based experiential learning environment and automatic story generation. The proposed system outputs the user's game experience as a text history. We consider this game as a learning experience accompanied by real-time emotions such as joy and surprise in an environment that allows various simulated experiences in a virtual space. The system used in this research was developed as a prototype for experiments to investigate the effect of the user's emotion and action history on game-based learning. Game-based learning (Kapp, 2012; Plass et al., 2020) is a method of learning while playing a game. Children nurture sociality and cognitive and emotional development through various games such as hide-and-seek and tag. In this game, we provide users with a learning experience of using a magical tool that can extract the contents of any object in a virtual space. The game is intended for learning to reflect on one's experience with the system. The user can later understand how he/she felt during the experience. For example, if a user stole something because no one was watching, he/she may recognize a character flaw or his/her lack of ethics. We predict that a learner's later review of this history, after a learning experience through the game, will be a learning aid. Therefore, we examine whether the history of the learner's actions and emotions in real time during learning helps the learner's review.

## Game-Based Story Generation System Using Player's Emotions

**Figure 1:**
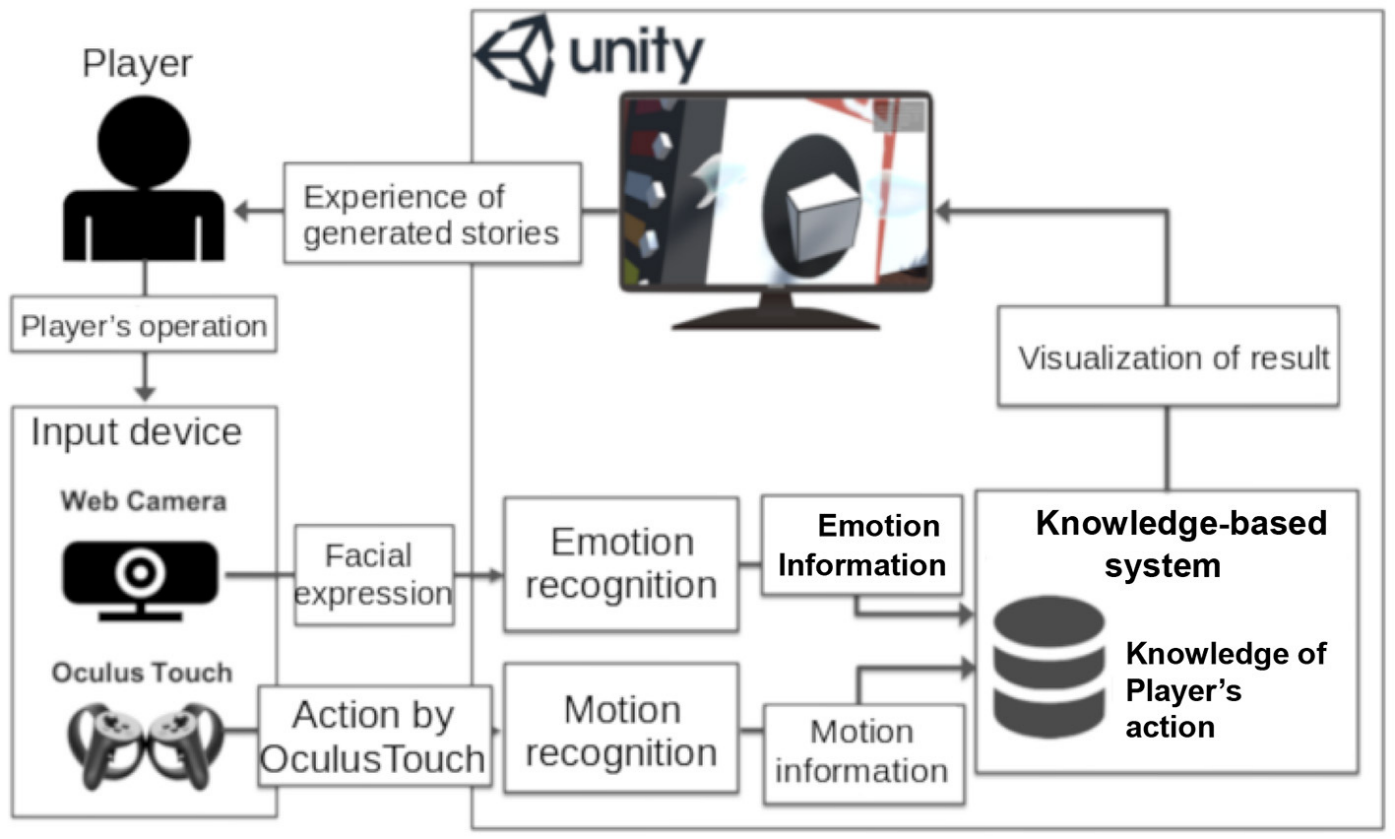
Overview of the proposed story generation system.

The proposed system, shown in Figure 1, provides game-based story generation using the player's real-time emotions and actions. It performs emotional recognition in real time by using a webcam to acquire facial images of the player. Specifically, the player uses the Oculus Touch, and the system classifies the player's actions by tracking the movement of his/her hand. We focus on emotion and behavior because previous studies have shown that body movement conveys emotion (Bianchi-Berthouze and Isbister, 2016). The system uses facial expressions for emotion recognition and Oculus Touch actions for motion recognition. Once the story generation system recognizes a particular action or emotion, it deploys a certain environmental action by using a knowledge-based system.

**Figure 2:**
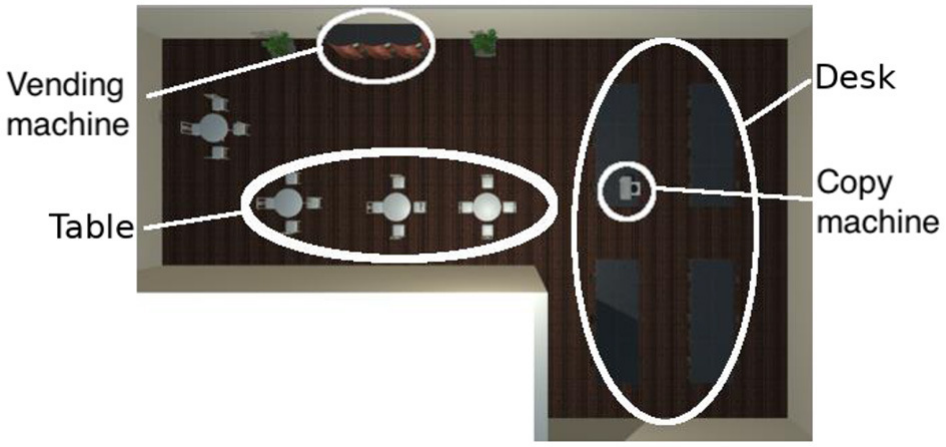
Bird's-eye view of the virtual space in the system, which represents an office and a break room.

The virtual space in the proposed system resembles a closed space found in everyday life, consisting of an office and a break room. The office and break room are not separated by a wall, so the player can move freely between the two areas. Figure 2 shows a bird's-eye view of the virtual space. The office has a desk and a copy machine, and the break room has a vending machine.

The system was inspired by a short movie called “The Black Hole^1^.” In the movie, the black hole was a mysterious sheet of paper printed by a company employee in an office. It was a “magical tool” that enabled human hands to pass through objects and take other objects inside. For example, when the black hole was placed on a vending machine, the employee's hand could pass through the front of the machine and pick up items inside. The employee cunningly used the strange paper to take snacks from the vending machine for free. He then thought of using this magical tool to pass his hand through a door and steal something that was normally inaccessible.

In our virtual space, the player can act upon objects of interest. To have players experience phenomena that do not occur in reality, they can use a “magical tool” to take objects from among a set of target objects. The magical tool consists of a black circle of paper, which is used to take objects by touching the desk or vending machine. Table 1 lists these interactive objects. For example, if the player touches the desk, he or she will take a document, a book, or a snack according to the player's emotion, such as “fear,” “anger,” or “joy”.

**Table 1:** List of interactive objects in the system.

**Object**	**Internal object**	**Detected emotion**
Desk	Document	Fear, disgust
	Book	Anger, sadness, surprise
	Snack	Joy, contempt, none
Vending machine	Cola	Fear, disgust, sadness, surprise
	Money	Joy, anger, contempt
	Cockroach	None

**Figure 3:**
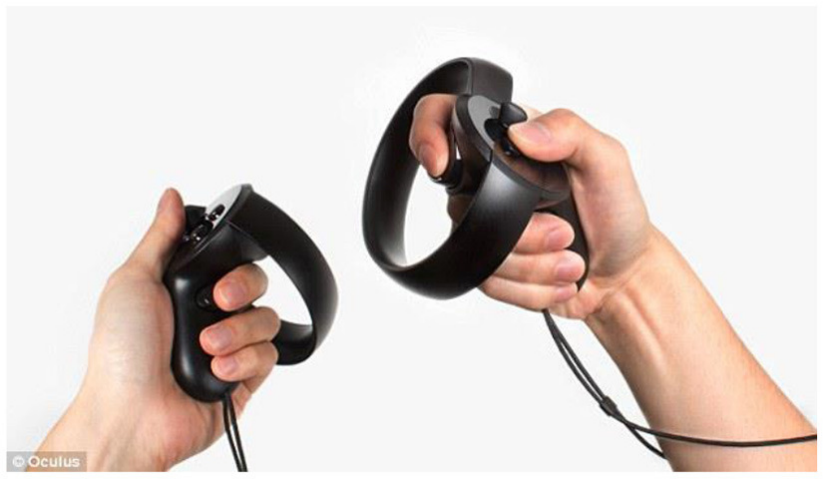
Oculus touch.

**Figure 4:**
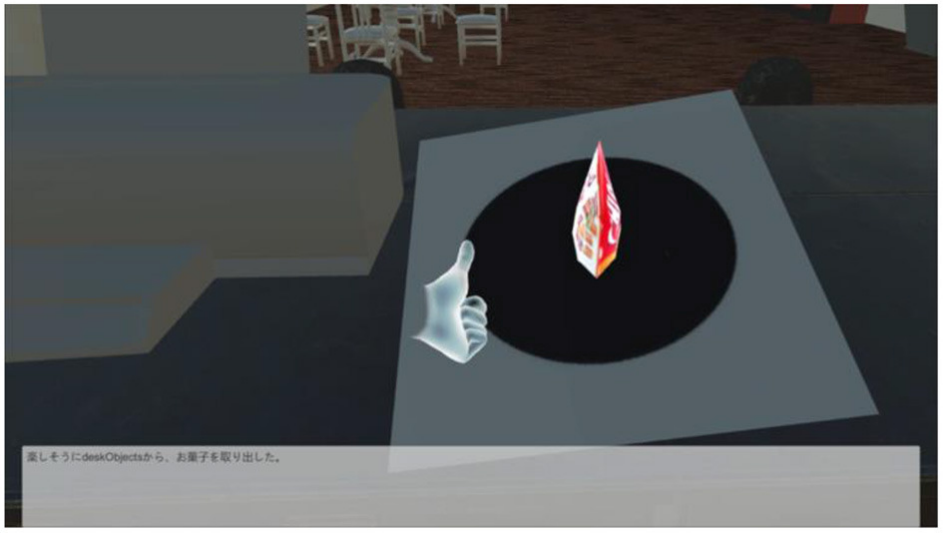
Example of player operation: taking a snack from the desk by using the virtual hand. Text: “You happily took the snack out of the vending machine”.

**Figure 5:**
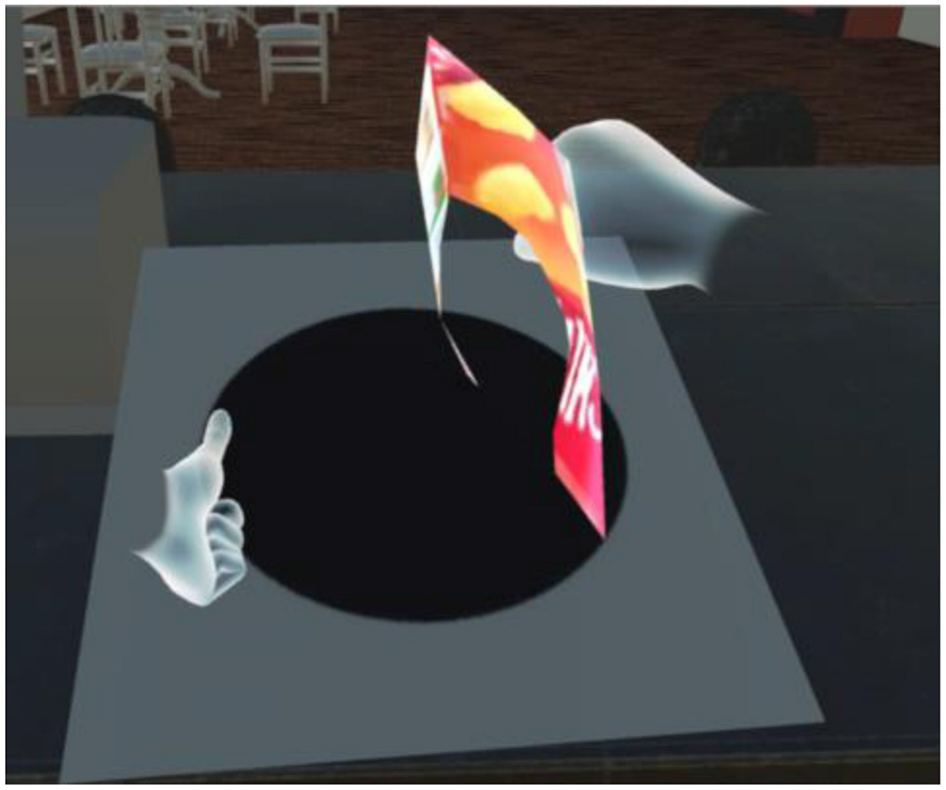
Example of player operation: eating a snack by using the virtual hand.

**Figure 6:**
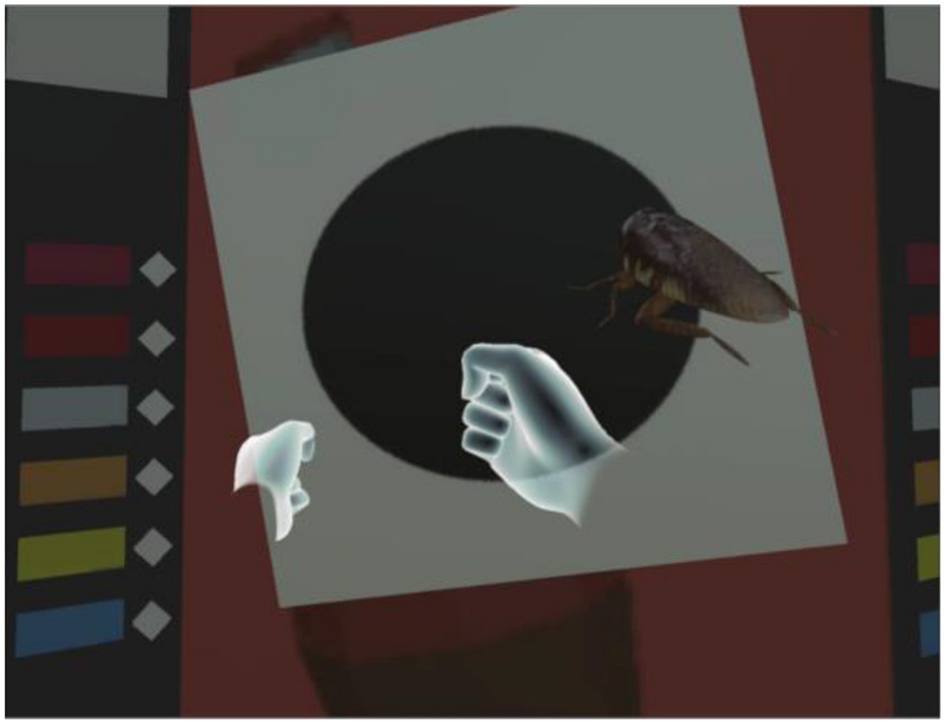
Example of player operation: taking a cockroach from the vending machine by using the virtual hand.

**Figure 7:**
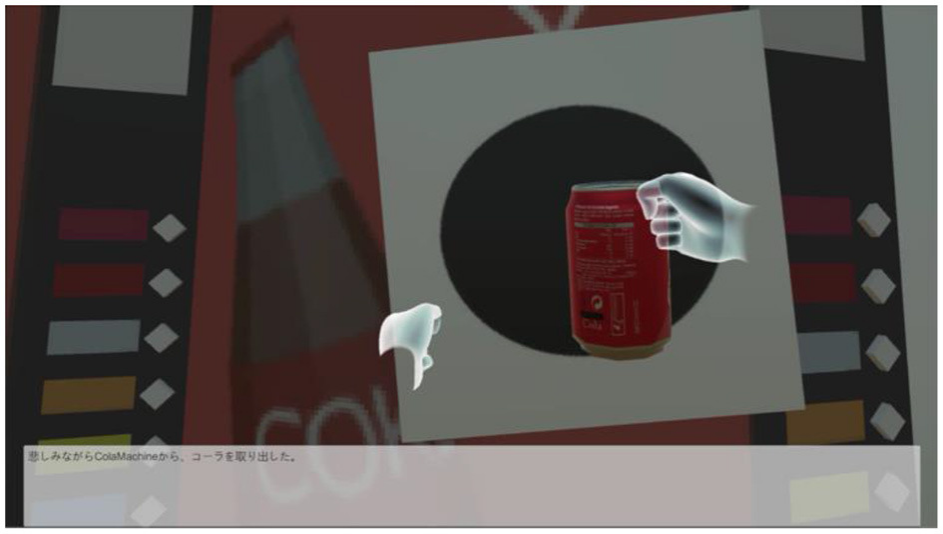
Example of script generation and visualization. Text: “You nervously took the cola out of the vending machine”.

In our system, the player uses the Oculus Touch, shown in Figure 3, to “take out,” “throw,” “put back,” “get,” and “eat/drink”. As shown in Figures 4, 5, the player operates the tool and experiences emotions at the same time, and then the system generates a story. A virtual hand is displayed on the screen, and when the user operates the Oculus Touch, the virtual hand moves. For example, the player can use the tool on the desk to take out a snack and eat it. Figure 4 shows the operation of taking a snack from the desk, and Figure 5 shows the operation of eating the snack. Figure 6 shows a situation in which the player takes a cockroach out of the vending machine, is surprised, and throws it away. Figure 7 shows a situation in which the player takes a cola out of the vending machine, along with an example of a generated script.

Through this content in the system, the player can try an experience that is impossible in real life, by using the magical tool to extract anything inside an object in the virtual space. Thus, players can learn by trial and error in a virtual space through an experience that they do not normally have.

### Motion Recognition

The Oculus Touch is a controller used for motion recognition. It uses two sensors to track its position in a three-dimensional virtual space. As the two sensors respond to the controller's movements, the virtual space displays a virtual hand that moves in the same way as the player's actual hand. The Oculus Touch has three input formats: button, analog stick, and trigger. In this system, we did not use a head-mounted display, because the system had to acquire the player's facial expressions. The system recognizes the action of holding the Oculus Touch trigger. If an object is located at the hand position, the player can hold the object. If the player releases the trigger while holding the object, the virtual hand releases the object.

### Emotion Recognition

The system performs emotion recognition in real time by using Affdex with the Facial Action Coding System (FACS; Ekman and Friesen, 1977). FACS encodes expressions of emotion by combining action units (AUs), which are the smallest units of visually distinguishable facial expressions. Affdex makes 20 predictions per second. The system uses Affdex and FACS to acquire seven types of emotions: joy, fear, disgust, sadness, anger, surprise, and contempt. We assume that if a special sensor device is used to acquire emotions, the player may be tense and unable to exhibit accurate emotions. Therefore, this system acquires player emotions *via* a camera.

One previous study (Magdin and Prikler, 2017) tested the accuracy of emotion recognition with Affdex. That study found that emotions can be recognized with high accuracy from in front of the face at a distance between 20 and 750 cm.

The advantage of using facial information is that the player can use the system freely, without wearing sensors (Kotsia et al., 2016). The system outputs scripts according to the player's emotion and action. It uses 30 predictions of 1.5 s each to determine the correct action. Affdex continuously stores the scores for each of the seven emotion types at a rate of ~30 times per second. When the player performs an action, the system sums the scores for each emotion type from the 30 most recent observations. It then determines which emotion was most strongly expressed and was the player's true emotion.

### Text Generation

The system produces scripts by combining the following: (1) the action performed with the Oculus Touch, (2) the player's recognized emotion, (3) the object acquired in the virtual space, and (4) the player's emotion information according to the object. The system has two types of text: one type generated from these four pieces of information, and another type generated automatically by the system. The first type is created by incorporating the four pieces of information into a sentence structure. Rows 8–13 in Table 2 give examples of the generated sentences.

**Table 2:** Example of a generated story.

1	You had been sleeping for some time because you were tired from work.
2	You woke up at your own workplace with no one around.
3	You noticed that the copier was moving, and you went to the front of the copier.
4	A black circle was printed on a piece of paper that emerged from the copier.
5	You felt a sense of strangeness and touched the black circle.
6	Suddenly, your hand was sucked into the circle.
7	You were surprised and removed your hand.
8	You took a book out of the desk with surprise.
9	You put the book back with surprise.
10	You took documents out of the desk with a lack of interest.
11	You put the documents back with a lack of interest.
12	You took a book out of the desk with surprise.
13	You put the book back with surprise.
14	You were holding the paper in your hand, but you did not feel like doing anything.
15	You decided to spend the day at the office.
16	In the morning, the paper was back to its original state, and you told your colleagues what you had experienced.

As an example of sentence generation, suppose that the system generated and displayed the sentence shown in Figure 7: “You nervously took the cola out of the vending machine.” The part corresponding to “You nervously” was generated because the system detected the emotion “fear.” Table 3 summarizes the outputs when each emotion is detected. As for the rest of the sentence, the term “vending machine” was the target object of the motion in the virtual space, and “cola” was obtained from knowledge of the vending machine object. Finally, the action “took out” was determined from the acceleration of the Oculus Touch and the part of the character that touched the object in the virtual space. This was used to determine the action result and its output, which was modified according to the emotion. Table 3 also lists the possible actions determined by the system, i.e., taking, throwing, putting, getting, eating, and drinking.

**Table 3:** Words generated from player emotions and actions.

**Recognized emotion**	**Generated word**	**Action by oculus touch**	**Generated** **word**
Joy	Happily	Hold trigger and pull hand	Took out
Fear	Afraid, nervously	Release trigger while shaking	
Disgust	Disgustingly	Swing controller	Threw away
Sadness	Sadly	Release trigger	Put back
Surprise	With surprise	Bring object to character's body	
Contempt	With contempt		Got
None		Bring object to character's head	Ate/drank

In this system, the story generation follows a dramatic narrative structure. The system provides the introduction and ending by using the given sentences listed in rows 1–7 and 14–16, respectively, in Table 2. The selected story ending differs depending on the player's actions. Three endings are available: (1) the player obtains three objects, (2) the player eats three foods, or (3) the player does nothing for 5 min.

Thus, the system visualizes and displays the results obtained by the knowledge-based method from the acquired actions and emotions. Here, we explain the sentence generation process using the sentences in Table 2. Rows 1–7 list introduction sentences that are displayed in the text space at the bottom of the screen so that the player can understand the instructions. For rows 8, 10, and 12, the player used the magical tool to “take out” an object from the desk; therefore, the taken internal object corresponds to the emotion determined from the knowledge of the target external object (i.e., the desk). For rows 9, 11, and 13, the player “put back” the object. The story ends when a specific action is not performed a certain number of times within 5 min. For rows 9, 11, and 13, the player acted after obtaining the object. The story ended when this eating action was performed three times. As with the action of eating, the system also ends the story if the player acts to obtain an object a certain number of times. Finally, rows 14 to 16 are ending sentences provided by the system. Once the system decides the story's ending, it darkens the screen and outputs the ending.

## Pilot Experiment

The purpose of this experiment was to evaluate the sentences generated by the system and its effectiveness. We thus evaluated the system's fun, operability, and future usefulness. In addition, we used the semantic differential (SD) method and a questionnaire to evaluate the fun and naturalness of the generated stories.

We recruited 18 male students majoring in computer science, whose ages were from 22 to 25, and we gave them a reward after their participation in the experiment. None of the students was familiar with any system equivalent to this one. We mentioned that the experiment could be stopped at any time, but no one chose to stop, so all subjects were included in the results here. The system was developed and evaluated as a prototype to investigate the use of action history with emotional expressions for review in learning. To familiarize themselves with the operation of the Oculus Touch, the subjects first experienced a tutorial. The tutorial had simple objects such as a cube, and the subjects practiced performing all the operations in Table 3. To enable the subjects to enjoy the system's content, we did not explain the magical tool in the tutorial. Instead, we let the subjects experience the tool directly in the system by explaining it to them orally and through the content.

For experimental material, we created a questionnaire to evaluate the stories generated by the system. The questionnaire addressed both the system and the generated narrative. Table 4 lists the items in the questionnaire. For each item, the subjects used a five-point scale consisting of “strongly disagree,” “disagree,” “unsure,” “agree,” and “strongly agree,” except for one item, Q3. In that case, Q3 was evaluated with three responses: “a story with emotion information,” “nothing,” and “a story without emotion information.” These questionnaire items also allowed free responses.

**Table 4:** List of questionnaire items.

	**Questionnaire content**	**Item**
Q1	The generated story is interesting.	5-point scale
Q2	The generated story is natural.	5-point scale
Q3	Between a story with emotion information and a story without it, which one gives you a better image of the situation or flow of the scene?	2-point scale
Q4	The tutorial is easy to understand.	5-point scale
Q5	The story generation phase is easy to operate.	5-point scale
Q6	I could easily make a story.	5-point scale
Q7	The story was made from my own actions.	5-point scale
Q8	I want to make a story using the system again.	5-point scale
Q9	I want to make a story in another situation (virtual space).	5-point scale

For additional experimental material to measure the subjects' impressions of the whole generated story, we also created a questionnaire using the SD method. Table 5 lists the scales for the SD method and their positions on the questionnaire. The items of positive impression on each scale were described using net betting. The choices for the scale were “very negative,” “rather negative,” “fairly negative,” “neither,” “fairly positive,” “rather positive,” and “very positive.”

**Table 5:** List of SD method scales.

**Scales of SD method**	
Natural	Unnatural
Intellectual	Emotional
Funny	Boring
Varied	Monotonous
Absurd	Sensible
Induced boredom	Induced concentration
Dynamic	Static
Unique	Common
Active	Passive
Hard to read	Easy to read

The following outlines the experimental method. The experiment time per subject was about 30 mins.

1) Explanation of the experiment
2) System tutorial
3) Story generation by the system
4) Confirmation of the generated story
5) Questionnaire using the SD method
6) Questionnaire on the system and story

We first explained the details of the experimental procedure, including the system's purpose. We then explained what kinds of operations could be done, by using both verbal and video instructions. After that, the subject was asked to use the system.

The flow of system use consisted of 5 mins for the tutorial and 5 mins for story generation. The time taken to generate a story changed the story's flow depending on the actions taken in the virtual space. Therefore, the system was set to end the story when its branching condition was satisfied. After experiencing the system, the subject responded to the SD method questionnaire and the questionnaire on the system and the generated story. The purpose of the SD method questionnaire was to obtain the subjects' intuitive impressions of the generated story, *via* a scale between pairs of adjectives. Note that we instructed the subjects to avoid responding with “neither” as much as possible, unless they could not determine the position between the two adjectives. The experiment ended once the questionnaires were complete.

As listed in Table 6, the questionnaire results were scored from 1 to 5 points for each item, ranging from “strongly disagree” to “strongly agree” for the items other than Q3. For tabulation of Q3, the response of “a story with emotion information” was counted as 1 point, while the other responses were each counted as 0. Then, the mean and standard deviation were calculated as descriptive statistics.

**Table 6:** Questionnaire items and scoring.

**Item**	**Score**
Strongly disagree	1.00
Disagree	2.00
Unsure	3.00
Agree	4.00
Strongly agree	5.00

As listed in Table 7, the SD method results from “very negative” on the left to “very positive” on the right were scored from −3 to 3 points, respectively, and then tabulated. For this scoring, if the mean value for an element of the SD method questionnaire was a negative number, it meant that the item on the left side was being evaluated. Similarly, a positive mean value meant that the item on the right was being evaluated. After tabulating the SD results, we calculated the mean and standard deviation as descriptive statistics and plotted a semantic profile. We also applied factor analysis by ProMax rotation of an unweighted least-squares method. All analyses used IBM SPSS Statistics 25.

**Table 7:** SD method items and scoring.

**Item**	**Position on the questionnaire**	**Score**
Very negative	Left	−3.00
Rather negative	Left	−2.00
Fairly negative	Left	−1.00
Neither	–	0.00
Fairly positive	Right	1.00
Rather positive	Right	2.00
Very positive	Right	3.00

## Experimental Results

The responses to each questionnaire item were calculated as a percentage of the total number of responses. Table 8 summarizes these percentages. A univariate chi-square test on these results showed that

**Table 8:** Questionnaire results.

		**Strongly agree**	**Agree**	**Unsure**	**Disagree**	**Strongly disagree**
Q1	Story is interesting	5%	61%	6%	22%	6%
Q2	Story is natural	0%	45%	11%	33%	11%
Q4	Tutorial is easy to understand	44%	28%	11%	17%	0%
Q5	Easy to operate	11%	39%	17%	33%	0%
Q6	Easy to make story	56%	33%	5%	6%	0%
Q7	Story made from my own actions	72%	11%	6%	11%	0%
Q8	Want to make story again	67%	16%	0%	17%	0%
Q9	Want to make story in another situation	72%	22%	0%	6%	0%
		Sentence with emotion information		Sentence without emotion information		
Q10	Scene imagination		94%		6%

Q1: Story is interesting (χ2 = 20.89, df =4, *p* < 0.05),
Q3: Scene imagination (χ2 = 14.22, df =1, *p* < 0.05),
Q6: Easy to make story (χ2 = 12.67, df =3, *p* < 0.05),
Q7: Story made from my own actions (χ2 = 21.56, df =3, *p* < 0.05),
Q8: Want to make story again (χ2=9.00, df = 2, *p* < 0.05),
and Q9: Want to make story in another situation (χ2 = 13.00, df = 2, *p* < 0.05) were significant at the 5% level. No significant differences were found for the other items.

**Figure 8:**
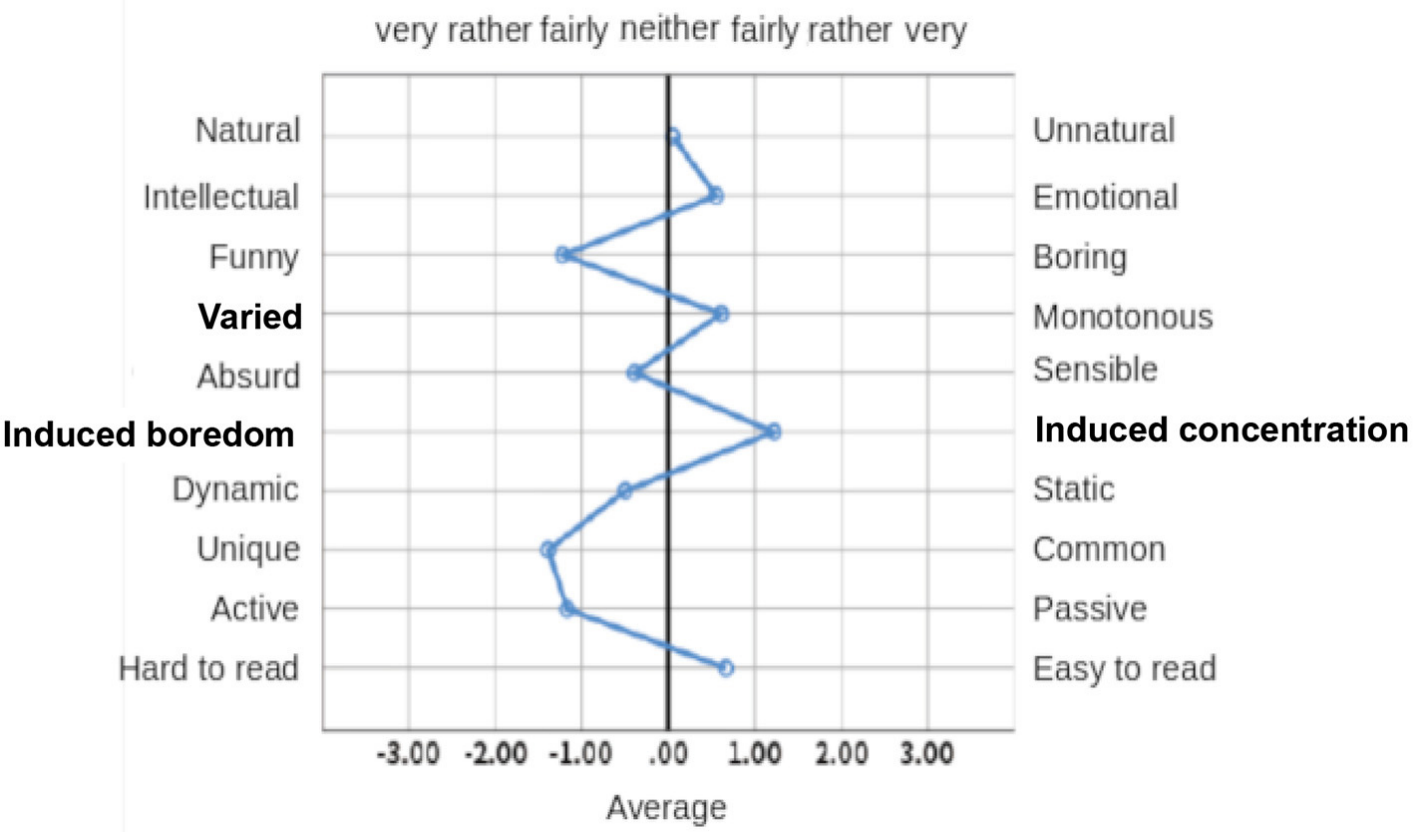
Semantic profiles.

Figure 8 shows the semantic profiles for the mean values of each scale. “Funny,” “absurd,” “dynamic,” “unique,” “active,” and other impressions were high, which suggests that the hands-on learning experience was enjoyable. As for the SD method results, Table 9 lists their descriptive statistics describing mean (M) and standard deviation (SD), and In addition, Table 10 lists the factor analysis results, while Table 11 summarizes the factor correlation matrix. In Table 10, numerical values with factor loadings <-0.4 or >0.4 are shown in bold to make it easier to understand the factors having a strong correlation with the extracted factors. Finally, the factor analysis results were used to perform factor interpretation, as listed in Table 12. From these results, we could extract three factors, namely, non-immersivity, non-novelty, and narrativity.

**Table 9:** Descriptive statistics for each scale of the SD method.

**Scale of SD method**		***M***	***SD***
Natural	Unnatural	0.06	2.00
Intellectual	Emotional	0.56	2.18
Funny	Boring	−1.22	1.96
Various	Monotonous	0.61	2.45
Absurd	Sensible	−0.39	1.91
Induced boredom	Induced concentration	1.22	1.83
Dynamic	Static	−0.50	2.12
Unique	Common	−1.39	1.61
Active	Passive	−1.17	1.76
Hard to read	Easy to read	0.67	2.00

**Table 10:** Factor loading list.

**Adjective**		**Factor 1**	**Factor 2**	**Factor 3**
Various	Monotonous	**0.929**	−0.279	0.261
Dynamic	Static	**0.922**	−0.024	−0.241
Funny	Boring	**0.550**	0.290	−0.043
Became bored	Concentrated	–**0.522**	–**0.439**	0.050
Intellectual	Emotional	−0.317	−0.199	−0.240
Active	Passive	0.063	**0.903**	0.658
Unique	Common	0.091	**0.658**	−0.042
Natural	Unnatural	0.134	–**0.542**	0.037
Hard to read	Easy to read	−0.168	0.233	**0.732**
Absurd	Sensible	0.163	−0.228	**0.591**
Load sum of squares after rotation		2.722	2.322	1.098
Contribution rate (%)		31.036	15.409	10.435
Cumulative contribution rate (%)		31.036	46.445	56.880

**Table 11:** Factor correlation matrix.

**Factor**	**1**	**2**	**3**
1	1.00	0.277	−0.055
2	0.277	1.00	0.077
3	−0.055	0.077	1.00

**Table 12:** Factor interpretation.

**Factor**	**Adjectives**	**Sum of squares of loading after rotation**	**Factor interpretation**
1	Monotonous, static, boring, became bored	2.722	Non-immersivity
2	Became bored, passive, common, natural	2.322	Non-novelty
3	Easy to read, sensible	1.098	Narrativity

## Discussion

In this study, we proposed a system that records one's emotions at the time of interaction with a virtual environment during experiential learning using a virtual space. This game-based system generates stories that includes the user's actions and emotions. We conducted an experimental evaluation of the system by subjects. The results showed that the generated stories were of interest to the subjects and were relatively easy to generate. Among the free responses for Q7 in the questionnaire, “The story was made from my own actions,” subjects answered with “It was interesting that my actions were written and read objectively” and “It was fun to look back on my actions.” Therefore, we believe that generation of action-based stories in a virtual space can appeal to users. Additionally, we found that the subjects enjoyed playing, because there was a significant difference in the questionnaire item Q8: “I want to make a story again.” Thus, players should be able to generate stories with various patterns by playing many times.

Unfortunately, the subjects were confused because the operation practice in the tutorial was not the same as the operation method in the virtual space. They needed to perform non-tool actions such as grabbing, throwing, and eating, but some subjects did not know how to perform those actions. Hence, the tutorial should allow the player to practice all the actions.

The “narrativity” factor obtained in the SD method questionnaire can be interpreted as showing that the generated narratives were consistent throughout. Therefore, the stories were considered to have a natural flow. The factor of “non-immersivity,” however, can be interpreted as indicating that the narrative generated by the player's actions did not feel immersive. This suggests a need to improve the virtual space to allow more types of actions. Lastly, the “non-novelty” factor indicates that there was not much change in the scene or behavior. In other words, because the system's virtual space was an average office, it may have lacked novelty. As there was a significant difference in the questionnaire item, “I want to make a story in another situation,” creation of a new scene may lead to improvement in the system's “novelty.”

The system records the history of the player's actions and emotions, so if a player's own game experience is interesting, it will be an interesting story for other people. If we think of this as a record of the learning experience in the game, an impressive record with emotions will provide support for learning. The results suggest that historical information, in which a learner's actions and emotions are recorded in real time, is useful after the learner has experienced a game, because it enables the learner to remember his/her own experience. There is not much previous research on effective use of historical information in game-based learning. We believe, however, that it is effective to use the history of actions and emotions recorded in real time for learning.

From the questionnaire, we found that it was easier for the subjects to imagine the scene by adding emotion information. The reason was that “because my emotions are recorded, it is easy to remember what I am doing.” There was also an opinion that “I hate insects, but I can picture throwing them away later, so I can remember them.”

For the same kind of information, the amount of information is different between situations with and without emotional expressions; with emotions, the descriptions are more specific, and the actions are more easily recalled. In the example above, rather than simply saying “throwing away insects, “it would be better to say” throwing away insects while feeling bad,” so that the emotions of the moment can be understood and the reasons for them can be recalled.

Various studies have shown that humans are more likely to remember emotional words than neutral words. For example, emotional words were remembered significantly more than neutral words when sentences with emotional evocations were compared to sentences with neutral words (Brierley et al., 2007). In a study that manipulated the valence of the stimuli (semantically relevant or irrelevant, positive, negative, or neutral words) and the type of encoding task (familiarity or emotional focus), regardless of semantic relevance, a memory advantage was revealed for emotional words (positive or negative words; Ferré et al., 2015). Also, high emotional contexts were remembered more than low emotional contexts (Schmidt, 2012).

We believe that the learning process can be strengthened by having the user experience it by himself/herself, keeping a history of the emotions at that time, and reviewing the text with the recorded actions and emotions again after the learning process. In addition to the fact that the user's own experience has already been retained in his/her memory, we believe that the emotional words used in the review will be easily retained in the memory, which will further strengthen it.

With a system such as the one used in this study, we expect that historical information, in which learners' actions and emotions are recorded in real time, will have a positive effect on learning because learners will be able to recall their experiences after experiencing the game. There is a possibility that experiential learning through games using virtual spaces, such as the one used in this study, will become widespread in the future. In such cases, it will be necessary to examine the learning effects of using historical information with emotions. Therefore, we believe that the results and discussions in this study will be useful for experiential learning using virtual spaces.

In the future, we would like to continue our research on the effective use of history information in game learning, and to investigate the use of real-time recorded history of actions and emotions for learning.

We have considered the effect of using action history with emotional expressions for review in learning. In this research, we investigated experiential learning content, but we have not yet conducted experiments on general learning support systems, so further investigation will be necessary. Furthermore, we need to investigate what kinds of granularity and timing are effective in using emotional expressions for learning content.

## Conclusion

In this study, we developed a game-type story generation system for experiential learning, which automatically generates scripts in real time by using emotions and actions in game-based learning using virtual space. This system provides players with a virtual world and virtual tools that they manipulate using a hand controller and a display. The system recognizes the player's emotions in real time from his/her facial expressions and outputs reactions based on those emotions by using a knowledge-based system. The story generation system then outputs scripts based on the emotions and action history.

We conducted an experiment with university students as subjects to evaluate this system. The system was able to generate stories that subjects found interesting based on the player's experience in the game by using the player's action history and emotions. The results suggest that historical information, including learners' real-time actions and emotions, can be useful for review in learning.

## Data Availability Statement

The original contributions presented in the study are included in the article/supplementary material, further inquiries can be directed to the corresponding author/s.

## Ethics Statement

Ethical review and approval was not required for the study on human participants in accordance with the local legislation and institutional requirements. The patients/participants provided their written informed consent to participate in this study.

## Author Contributions

All authors listed have made a substantial, direct, and intellectual contribution to the work and approved it for publication.

## Conflict of Interest

The authors declare that the research was conducted in the absence of any commercial or financial relationships that could be construed as a potential conflict of interest.

## Publisher's Note

All claims expressed in this article are solely those of the authors and do not necessarily represent those of their affiliated organizations, or those of the publisher, the editors and the reviewers. Any product that may be evaluated in this article, or claim that may be made by its manufacturer, is not guaranteed or endorsed by the publisher.

## Footnotes

^1^The Black Hole | Future Shorts https://www.youtube.com/watch?v=P5_Msrdg3Hk.
